# Dental Implant Osseointegration Inhibition by Nicotine through Increasing nAChR, NFATc1 Expression, Osteoclast Numbers, and Decreasing Osteoblast Numbers

**DOI:** 10.1055/s-0042-1758794

**Published:** 2022-12-27

**Authors:** Nina Nilawati, Widyastuti Widyastuti, Yoifah Rizka, Hansen Kurniawan

**Affiliations:** 1 Department of Periodontology, Faculty of Dentistry, Universitas Hang Tuah, Surabaya, Indonesia; 2 Department of Periodontology, Haji General Hospital, Surabaya, Indonesia

**Keywords:** dental implant, nicotine, nAChR, NFATc1, osteoclast, osteoblast, osseointegration

## Abstract

**Objective** The success of dental implants is determined by the osteointegration process. Many studies state that smoking cigarettes can inhibit osseointegration, but the inhibition mechanism is still unclear.

The aim of this study was to identify and analyze the effect of nicotine on the inhibition of dental implant osseointegration through the expression of nicotinic acetylcholine receptor (nAChR), nuclear factor of activated T cells cytoplasmic 1 (NFATc1), osteoclast, and osteoblast numbers.

**Materials and Methods** This study is an experimental study of 16 New Zealand rabbits, randomized across two groups. Group 1 (eight rabbits) was a control group, and group 2 (eight rabbits) was a treatment group. The treatment group was given 2.5 mg/kg body weight/day of nicotine by injection 1 week before placement of the implant until the end of research. Observations were made in the first and the eighth week by measuring the number of osteoblast and osteoclast by immunohistology test and the expression of nAChR and NFATc1 by immunohistochemistry test.

**Statistical Analysis** Data was analyzed using a one-way analysis of variance and Student's *t* -test. A *p* -value of < 0.05 was considered statistically significant.

**Results** Significant differences were found between the control and treatment groups ( *p* < 0.05). Results showed that nicotine increases the expression of nAChR and decreases the number of osteoblasts and the expression of BMP2 and osteocalcin.

**Conclusion** Nicotine inhibits the osseointegration of dental implants by increasing nAChR, NFATc1, osteoclast numbers, and decreasing osteoblast numbers.

## Introduction

Dental implants are the gold standard for the replacement of missing teeth. Many factors have been recognized as critical for the successful performance of the implants. One of the most important factors is osseointegration—the direct interactions between the implant and the tissues. The osseointegration depends on the osteoblastic activity enhancement around the implant that promotes direct union between the implant and the bone. The success rate of osseointegration of dental implants depends on many factors such as oral hygiene, operator skills, implant materials used, bone quality and quantity, occlusal load, lack of medical condition, and personal oral habits such as smoking. 1 2 Smoking is considered one of the major causes of the failure of dental implants. 3 4 5 6

Smoking is a high-risk factor and contributes to the causes of oral diseases, including periodontitis, which usually presents as alveolar bone resorption. 7 Nicotine is a major component of tobacco that regulates alveolar bone metabolism by increasing periodontal tissue attachment loss and alveolar bone absorption, increasing periapical disease, inducing peri-implantitis, and decreasing bone regeneration rate after tooth extraction or surgery. 4 8 9 Nicotine affects initial implant survival rates and early osseointegration. 10 11 12 The higher the duration and frequency of smoking, the higher the rate of marginal bone loss around the dental implant. 13 Nicotine contributes to an increase in osteoclastic activity that causes bone loss. The harmful effects of nicotine can be so detrimental that it affects wound healing and ultimately affects implant osseointegration. 14

Bone remodeling is a physiological process that involves a balance between bone formation and bone destruction regulated by osteoblasts and osteoclasts. 15 16 The activated T cell nucleus factor, cytoplasm 1 (NFATc1), which is the master transcription factor for osteoclast formation, can induce osteoclast differentiation in the absence of RANKL. 17 NFATc1-deficient osteoclast precursors cannot differentiate into osteoclasts, but ectopic expression of NFATc1 causes osteoclast differentiation. 18 Osteoclastogenesis is also regulated by nicotinic acetylcholine receptors (nAchRs). nAChRs have been identified in osteoblasts and osteoclasts cells that are known to be essential for maintaining bone homeostasis. 19

The mechanisms behind the effect of nicotine on dental implant osseointegration are not fully understood. Some studies have shown that nicotine inhibits bone metabolism, while others have shown a biphasic effect. 20 Therefore, the purpose of this study was to identify and analyze the effect of nicotine on the inhibition of osseointegration of dental implants on the expression of nAChR, NFATc1, osteoclast, and osteoblast numbers.

## Materials and Methods

### Preparation of Experimental Animals

This study was an experimental study that designed a posttest control group. Sixteen healthy New Zealand White male rabbits 3.5 to 5.0 months old and weighing 2.5 to 3.5 kg were acclimatized for 7 days. Rabbits were housed in cages and given the same standard food and water *ad libitum* before, during, and after treatment.

Rabbits were randomly divided into two groups, a control group and a treatment group, each consisting of eight rabbits. Each group was divided into two small groups, 1 week and 8 weeks. In the treatment group, nicotine was induced by injecting a dose of 2.5 mg nicotine hydrogen tartrate salt per kg body weight (BW) (Sigma-Aldrich, United Kingdom). The control group was not given a nicotine induction.

### Dental Implant Insertion

All rabbits were weighed to determine the required dose of anesthesia. All rabbits were anesthetized by intramuscular injection with a total dose of 0.15 mL xylazine per kg BW and 0.2 mL ketamil per kg BW.

Before placing the implant, the jawbone was marked and drilled into the bone to a depth of 7 mm at a speed of 1,200 revolutions per minute. An implant (SG, South Korea) with a diameter of 3.3 mm and a length of 7 mm was placed on the bone. Rabbits were injected with long-acting antibiotics (Medoxy-LA) at 0.1 mL/kg BW.

### Sample Collection

At weeks 1 and 8, after implant insertion, all rabbits were euthanized with 0.2 mL ketamine by the dose of 10 mg/kg BW. Osseointegration values were measured by examining the stability of the implant using the ISQ Osstell tool. The number range on the Osstell tool was 1 to 100. If the Osstell tool shows more than 55, it is considered to have osseointegration and no osseointegration when less than 55. Osteoclast and osteoblast counts were examined from the mandibular section using Meyer's hematoxylin and eosin staining (Sigma-Aldrich).

Expression of nAChR and NFATc1 was performed using immunohistochemical staining with monoclonal primary antibody anti-nAChR (LSBio, United States) and anti-NFATc1 (Sigma, United States) marked by a brown color in the cell cytoplasm. Expression of nAChR and NFATc1 from cells was calculated by observing 10 different fields of view under a microscope at 400× magnification.

### Data Analysis

Data on the number of osteoblasts and osteoclasts, nAChR, and NFATc1 expression are expressed as mean ± standard deviation and statistics by analysis of variance and Student's *t* -test using SPSS software package version 17.0. A *p* -value of < 0.05 was considered to show a statistically significant difference.

## Results

The effect of nicotine on osseointegration of dental implants was examined, and the results are shown in Table 1 . In the first week, there was no significant difference between the control and nicotine groups ( *p* > 0.005), but in the 8th week, the osseointegration value in the treatment group was lower than in the control group ( *p* < 0.005).

**Table 1:** The value of osseointegration at week 1 and 8

Variable	Time	Group ( *n* = 8)	Mean	SD	*p*
Osseointegration	Week 1	Control	51.38	0.744	0.997
		Nicotine	51.13	0.991	
	Week 8	Control	65.63	2.134	0.000
		Nicotine	52.13	2.031	

Abbreviation: SD, standard deviation.

Osteoclast numbers increased in the nicotine group at weeks 1 and 8 compared with the control group ( *p* < 0.005). Osteoblast numbers in the nicotine groups were decreased compared with the control groups at weeks 1 and 8 ( *p* < 0.005) ( Table 2 ). The results show that expression of nAChR and NFATc1 was higher in the 1st and 8th week nicotine groups than in the control group ( *p* < 0.005) ( Table 3 ).

**Table 2:** The number of osteoclasts and osteoblasts cells at week 1 and 8

Variable	Time	Group ( *n* = 8)	Mean	SD	*p*
Osteoclast	Week 1	Control	13.00	2.390	0.000
		Nicotine	18.63	4.104	
	Week 8	Control	10.50	1.773	0.000
		Nicotine	29.63	2.875	
Osteoblast	Week 1	Control	21.13	2.360	0.000
		Nicotine	10.50	1.770	
	Week 8	Control	27.88	2.950	0.000
		Nicotine	6.88	2.360	

Abbreviation: SD, standard deviation.

**Table 3:** The expression of nAChR and NFATc1 at week 1 and 8

Variable	Time	Group ( *n* = 8)	Mean	SD	*p*
nAChR	Week1	Control	5.38	2.667	0.000
		Nicotine	12.00	1.773	
	Week 8	Control	5.00	2.390	0.000
		Nicotine	18.50	2.976	
NFATc1	Week 1	Control	4.50	2.000	0.000
		Nicotine	11.25	3.240	
	Week 8	Control	3.50	1.690	0.000
		Nicotine	11.25	22.510	

Abbreviations: nAChR, nicotinic acetylcholine receptor; NFATc1, nuclear factor of activated T cells cytoplasmic 1; SD, standard deviation.

## Discussion

This study shows that nicotine inhibits the osseointegration of dental implants by decreasing osteoblast numbers and increasing osteoclast numbers. Nicotine exposure increased the expression of nAChR and also the expression of NFATc1.

After dental implant insertion, mechanical trauma and bone damage caused by inflammation stimulate bone cells to produce cytokines and growth hormones to repair the damage. Bone healing around the implant involves a cascade of cellular and extracellular biological events at the interface between the bone and the implant until the surface of the implant is covered with newly formed bone. This biological process is regulated by growth and differentiation factors released at the bone-implant interface. 21 22

In this study, we found that osseointegration was inhibited by nicotine induction. Nicotine is more likely to spread and has a negative effect on bone healing. The osseointegration process requires the recruitment of osteoblasts, their fixation, adhesion, diffusion, proliferation, and differentiation into osteoblasts that secrete extracellular matrix calcification on the implant surface. All of these cellular events are sensitive to local and systemic effects of nicotine. 23

Osteoblast activity was reduced in the experimental group compared with the control group. These results are consistent with those of Kim et al and Shibli et al who reported that nicotine suppresses the proliferation of osteoblasts. 10 24 Nicotine acts as a stimulant and inhibitor of bone metabolism. Nicotine reduces the activity of osteoblasts and affects the collagen that can be used to form the extracellular matrix. Nicotine can also induce microvascular obstruction and reduce blood cell proliferation that impairs healing areas after implant placement. 25 Nicotine also reduces macrophage proliferation and increases susceptibility to infection at the surgical site of implant placement. 26 Another study states that nicotine concentration alters the effect of nicotine on implant osseointegration. Low nicotine levels stimulate osteoblast proliferation, increased the expression of osteocalcin, type 1 collagen, and alkaline phosphatase, but high levels have the opposite effect. 13 Conversely, Pereira et al found that exposure to nicotine adversely affected osteoblast activity. 23

This study demonstrated that nicotine also promotes osteoclast formation. This finding is supported by Henemyre et al, who states that exposure to nicotine increases osteoclast differentiation and calcium phosphate absorption during bone regeneration. 27 Nicotine can affect osteoclast formation through several mechanisms. In contrast, Adler and Attia found that nicotine does not stimulate the formation of osteoclasts in rat bone marrow. 28

In this study, nicotine induction increased nAChR expression. nAChR has been identified in cells essential for maintaining bone homeostasis, such as osteoblasts and osteoclasts. 19 Recently, several studies have linked nAChR to the regulation of osteoclast formation. 29 30 31 32 nAChR agonists such as nicotine and carbachol choline have been shown to stimulate osteoclast formation. 29 33 34 In addition, nAChR antagonists block most of the effects of nicotine, suggesting that these changes are dependent on nAChR activation. 35

We also found that nicotine induction increased NFATc1 expression. NFATc1 is a master transcription factor of osteoclast regulators involved in osteoclast formation and osteoclast activation. 36 Induction of nicotine induces excessive osteoclast activity and bone resorption, impairing osseointegration of dental implants. RANK induces the NFATc1 gene in osteoclasts via transcription factors such as the nuclear factor kappa B and c-Fos. 33 The important role of NFATc1 in osteoclast differentiation is well documented by several studies. Inhibition of NFATc1 by cytochalasin Z11 inhibits RANKL-induced osteoclast formation. 37 Even NFATc1-deficient embryonic stem cells cannot differentiate into osteoclasts in response to RANKL. However, NFATc1-ectopic osteoclast precursors induce osteoclast differentiation in the absence of RANKL. 18

## Conclusion

Nicotine inhibits the osseointegration of dental implants by increasing nAChR, NFATc1, osteoclast numbers, and decreasing osteoblast numbers.
